# Integrative omics and experimental validation reveal METTL17 and SLC27A1 as biomarkers and potential therapeutic targets in chronic kidney disease

**DOI:** 10.3389/fimmu.2026.1724740

**Published:** 2026-02-13

**Authors:** Shiyun Ling, Kaifeng Xie, Penghui Chen, Lichang Liang, Xinyu Shi, Renfa Huang

**Affiliations:** Nephropathy Department, Shenzhen Hospital (Futian) of Guangzhou University of Chinese Medicine, Shenzhen, Guangdong, China

**Keywords:** chronic kidney disease, immune infiltration, macrophage polarization, mitochondria, single-cell sequencing analysis

## Abstract

**Background:**

Chronic kidney disease (CKD) remains a global health challenge characterized by high morbidity and mortality, yet its molecular mechanisms remain incompletely defined. Both mitochondrial dysfunction and macrophage polarization have been implicated in CKD pathogenesis, but the precise gene networks and cellular contexts driving these processes are poorly understood.

**Methods:**

An integrative analysis of bulk RNA sequencing, single-cell transcriptomics, and clinical validation was performed to identify key genes linking mitochondrial regulation and macrophage polarization in CKD. Differentially expressed genes were intersected with mitochondria- and macrophage-related gene sets, refined by machine learning, and assessed through functional enrichment, immune infiltration, and network analyses. Single-cell RNA-seq was applied to resolve cellular heterogeneity and ligand–receptor interactions, while experimental validation was carried out in CKD patient peripheral blood samples and a unilateral ureteral obstruction (UUO) mouse model using qPCR, immunohistochemistry, and Western blotting.

**Results:**

A total of METTL17 and SLC27A1 were identified as consistently dysregulated genes across datasets, with METTL17 downregulated and SLC27A1 upregulated. Single-cell analysis localized these alterations primarily to proximal tubular cells (PTCs) and smooth muscle cells (SMCs), where ligand–receptor signaling mediated pathogenic intercellular communication. Immune infiltration analysis revealed selective alterations in naïve B cells, activated NK cells, and γδ T cells, highlighting crosstalk between metabolic and immune pathways. Importantly, both genes showed positive correlations with serum creatinine in CKD patients (e.g., SLC27A1: r = 0.467, *P* = 0.038), underscoring their clinical relevance. Experimental validation in CKD patient peripheral blood samples (*P* < 0.01 for both genes) and in a unilateral ureteral obstruction (UUO) mouse model of renal fibrosis confirmed consistent dysregulation: METTL17 was significantly downregulated and SLC27A1 upregulated at both mRNA and protein levels (e.g., immunohistochemistry, *P* < 0.05). These alterations were spatially localized to proximal tubules and coincided with marked collagen deposition.

**Conclusion:**

This study identifies METTL17 and SLC27A1 as key mediators of CKD progression, bridging mitochondrial dysfunction, metabolic reprogramming, and immune imbalance. These findings provide a translational framework for developing biomarker-driven diagnostics and targeted interventions in CKD.

## 1 Introduction

Chronic kidney disease (CKD) is defined as persistent abnormalities in kidney structure or function, such as an estimated glomerular filtration rate (eGFR) < 60 mL/min/1.73 m² or albuminuria ≥ 30 mg/24 h, for at least three months, resulting in adverse health outcomes (1). CKD progression is closely associated with fibrosis, driven by inflammatory cell infiltration and aberrant extracellular matrix accumulation (2). Renal interstitial fibrosis is a hallmark of multiple common CKD subtypes. The extent of interstitial fibrosis serves as a robust morphological predictor of clinical outcomes (3), although its underlying mechanisms remain incompletely understood. Currently, CKD remains incurable, and its management primarily focuses on slowing disease progression and addressing associated cardiovascular complications (4). Therefore, elucidating the pathogenesis of CKD and identifying relevant diagnostic markers for targeted interventions may facilitate the development of more effective therapeutic strategies.

Macrophages are key components of the innate immune system, characterized by substantial heterogeneity and functional polarization (5). Mitochondria serve as the primary sites for ATP production and biosynthesis, and are essential for maintaining cellular metabolism and homeostasis (6). Disruptions in mitochondrial integrity, bioenergetics, or other metabolic and regulatory functions have been implicated in the pathogenesis of numerous diseases (7). Synergistic interactions between macrophage polarization and mitochondrial dysfunction exacerbate renal injury, inflammation, and fibrosis (8). In response to renal injury, M1 macrophages promote inflammation and tissue damage by secreting pro-inflammatory cytokines (IL-1, IL-6, IL-12, TNF-α), chemokines (e.g., IL-8), reactive oxygen species (ROS), and nitric oxide (NO) (9). The role of M2 macrophages in renal fibrosis, however, remains controversial due to their diverse and context-dependent functions. In acute or transient renal injury, such as acute tubular necrosis (ATN), M2 macrophages primarily exert anti-inflammatory effects, facilitating epithelial repair and tubular regeneration (10). By contrast, in chronic kidney disease, M2 macrophages often promote fibrosis through macrophage-to-myofibroblast transition (MMT) (11). Mitochondrial dysfunction and disrupted homeostasis in CKD impair energy metabolism and elevate mitochondrial-derived reactive oxygen species (mROS) production, thereby exacerbating renal injury and inflammation (12). Although evidence suggests that macrophage polarization and mitochondrial dysfunction are critical drivers of CKD progression, the molecular mechanisms remain insufficiently characterized and are likely mediated through specific gene regulatory networks (13).

Single-cell RNA sequencing (scRNA-seq) enables resolution of cellular heterogeneity at single-cell resolution by integrating reverse transcription, cDNA amplification, and library construction, thereby allowing high-throughput transcriptomic profiling of thousands of cells (14, 15). In kidney research, scRNA-seq has greatly facilitated the characterization of cellular heterogeneity, gene expression signatures, and molecular dynamics during renal disease progression, advancing diagnostic precision and therapeutic discovery (16, 17). Recent advances in single-cell RNA sequencing have enabled in-depth characterization of cellular heterogeneity within disease microenvironments, revealing distinct cellular subpopulations with specialized pathogenic functions and complex interaction networks (18). A seminal study identified a pro-inflammatory fibroblast subpopulation in CKD, characterized by SFRP1+FAP- expression and the absence of COL1A1, termed CXCL-iFibro (19). This subpopulation represents an early stage of myofibroblast differentiation and actively recruits macrophages, directing their polarization toward a FOLR2+ phenotype. In turn, macrophages activate the WNT/β-catenin signaling pathway, promoting the differentiation of CXCL-iFibro into ECM-secreting myofibroblasts and establishing a positive feedback loop that accelerates disease progression (19). Therefore, elucidating the regulatory networks of key biomarkers within specific cell subpopulations-such as renal tubular epithelial cells and smooth muscle cells-through integrated single-cell transcriptomic analysis is critical for this study.

In this study, transcriptomic and single-cell datasets were integrated to identify differentially expressed genes (DEGs) in CKD, which were intersected with mitochondrial-related genes (MRGs) and macrophage polarization-related genes (MPRGs) to obtain candidate targets. Key genes were further filtered using machine learning algorithms, validated across datasets, and assessed by receiver operating characteristic (ROC) curve analysis. Functional enrichment, chromosomal localization, regulatory network construction, and immune cell infiltration analyses were then performed. Additionally, scRNA-seq data provided insights into cellular heterogeneity and revealed the distribution of key gene expression among distinct subpopulations. Finally, expression of candidate genes was validated in clinical peripheral blood samples and in a unilateral ureteral obstruction (UUO) mouse model. This integrative multi-omics approach highlights mitochondrial- and macrophage-associated gene signatures and provides novel insights into the pathogenesis and potential therapeutic targets of CKD.

## 2 Methods

### 2.1 Data collection

The two transcriptome datasets were retrieved from the Gene Expression Omnibus (GEO) database (https://www.ncbi.nlm.nih.gov/geo). The GSE66494 dataset (platform: GPL6480) contains 53 kidney tissue samples from CKD patients and 8 normal kidney tissue samples, which were used as the training set. The GSE180394 dataset (platform: GPL19983) contains 43 kidney tissue samples from CKD patients and 9 normal kidney tissue samples, which were used as the validation set. A single-cell dataset, GSE171314 (platform: GPL20795), contains 4 kidney tissue samples from CKD patients and 1 normal kidney tissue sample. In the Molecular Signatures Database (MSigDB) (https://www.gsea-msigdb.org/gsea/msigdb), the keyword “Macrophage Polarization” was searched, and a total of 35 MPRGs were collected. Additionally, a total of 2,030 MRGs were obtained from the MitoCarta database (https://www.broadinstitute.org/mitocarta/mitocarta30-inventory-mammalian-mitochondrial-proteins-and-pathways) and the Gene Set Enrichment Analysis database (GSEA) (http://www.gsea-msigdb.org/gsea/index.jsp).

### 2.2 Identification of candidate genes

Differentially expressed genes (DEGs) between CKD and control samples in GSE66494 were identified using limma (v3.58.1) (20) with *P* < 0.05 and |log_2_Fold change (FC)| > 0.5. Volcano plots and heatmaps highlighting the top 10 up- and down-regulated DEGs were generated with ggplot2 (v3.5.1) (21) and pheatmap (v3.5.1) (22).For pathway-level macrophage polarization activity, single-sample GSEA (ssGSEA) was performed using GSVA (v1.50.0) (23) to compute MPRG scores per sample; group differences were evaluated with the Wilcoxon rank-sum test (*P* < 0.05) and visualized by ggplot2.To identify co-expression modules associated with macrophage polarization, WGCNA (v1.72-5) (24) was applied to GSE66494. Samples were hierarchically clustered using Euclidean distance to detect and remove outliers; the soft-thresholding power was chosen to approximate scale-free topology (R² ≈ 0.90 with near-zero mean connectivity). Adjacency and topological overlap matrices were computed to construct the gene dendrogram; dynamic tree cutting (minModuleSize = 50) was used to define modules. Module eigengenes were correlated (Spearman) with ssGSEA-derived MPRG scores using psych (v2.4.3) (25); genes within the most positively and negatively correlated modules (|cor| > 0.3, *P* < 0.05) were designated module genes. Module membership (MM) and gene significance (GS) were obtained via geneModuleMembership and geneTraitSignificance.Candidate genes were defined as the intersection of DEGs, MRGs, and module genes, computed with ggvenn (v0.1.10) (26).

### 2.3 Enrichment analysis of candidate genes and construction of protein-protein interaction network

Functional enrichment of candidate genes was performed with clusterProfiler (v4.10.1) (27) for Gene Ontology (GO: BP, CC, MF) and KEGG pathways (*P*.adjust < 0.05). GO terms were ordered by adjusted P-values, and the top 15 per category were visualized. To explore physical and functional interactions, STRING (http://www.string-db.org/; interaction score > 0.4) was queried to build a PPI network, which was visualized in Cytoscape (v3.10.2) (28). Nodes without reported interactions were omitted from the network display.

### 2.4 Identification of key genes

Feature selection in GSE66494 was performed using LASSO with glmnet (v4.1-8) (29). Perform 20-fold cross-validation using the cv.glmnet() function, employing binary classification bias as the model performance metric. This function automatically generates a sequence of λ values, recording the key parameters lambda.min and lambda.1se. Lambda.min corresponds to the λ value yielding the smallest cross-validation error, whilst lambda.1se denotes the λ value of the minimal model whose error falls within one standard error of the minimum value. Feature selection in this study primarily relied upon lambda.min; genes retained at this lambda value were designated as feature genes. Diagnostic performance was assessed by ROC analysis (pROC v1.18.5) (30) in both GSE66494 and GSE180394; genes with AUC > 0.7 in both datasets were considered candidate key genes. Group-wise expression differences for these genes were then tested in both datasets, and genes showing concordant directionality and statistical significance (*P* < 0.05) were designated key genes for downstream analyses.

### 2.5 Functional enrichment and chromosomal localization of key genes

To infer biological programs associated with key genes, Spearman correlations between each key gene and all genes were computed in GSE66494 using psych. Genes were ranked by correlation coefficients, and GSEA (clusterProfiler; MSigDB c2.cp.kegg.v7.4.entrez.gmt) was performed (*P* < 0.05, FDR < 0.25, |NES| > 1). The top five enriched pathways per key gene were reported. Genomic positions of key genes were annotated and visualized with RCircos (v1.2.2) (31).

### 2.6 Immune infiltration analysis

Immune cell composition (22 leukocyte subsets) (32) was estimated in GSE66494 by CIBERSORT (v0.1.0) (33). Samples with deconvolution *P* > 0.05 were excluded. Group differences were evaluated using the Wilcoxon rank-sum test (*P* < 0.05). Spearman correlations were computed among differentially abundant immune cells and between key genes and these immune cells (|cor| ≥ 0.3 and *P* < 0.05), implemented in psych and visualized with ggplot2.

### 2.7 Molecular regulatory network and functional correlation analysis of key genes

Transcription factors (TFs) potentially regulating key genes were predicted via NetworkAnalyst (https://www.networkanalyst.ca/). miRNAs targeting key genes were predicted using miRWalk (http://mirwalk.umm.uni-heidelberg.de) (top 100 candidates by accessibility; shared miRNAs for both key genes were prioritized). TF–mRNA, miRNA–mRNA, and integrated TF–miRNA–mRNA networks were visualized in Cytoscape.GeneMANIA (http://genemania.org) was used to infer functionally related genes and interaction types (e.g., co-expression, physical interaction). Clinical relevance was evaluated using Nephroseq v5 (http://v5.nephroseq.org) by correlating key gene expression with renal function parameters (Spearman; threshold cor ≥ 0.30, *P* < 0.05). (Where applicable, multiple testing was controlled; exploratory correlations are interpreted cautiously).

### 2.8 Processing of the scRNA-seq data

Single-cell analyses for GSE171314 were performed in Seurat (v5.0.1) (34). Quality control filters were: 200 < nFeature_RNA < 6,000; percent.mt < 25%; nCount_RNA < 20,000. Data were normalized and scaled with NormalizeData and ScaleData. Highly variable genes (HVGs; n = 2,000) were identified using the vst method (FindVariableFeatures); the top 10 HVGs were labeled (LabelPoints). Principal component analysis (RunPCA) was followed by ElbowPlot and JackStraw to select significant PCs (*P* < 0.05). Cells were clustered (resolution = 0.6) and embedded by UMAP.Differentially expressed markers per cluster were identified with FindAllMarkers (min.pct = 0.25, logfc.threshold = 0.25). Cell identities were assigned using literature-curated markers (35) as primary and CellMarker (http://117.50.127.228/CellMarker/) as secondary reference (**Supplementary Table S1**). Marker representation was visualized with bubble plots, and UMAPs were colored by annotated cell types. AUCell (v1.20.0) (36) was used to compute per-cell activity scores for key genes (AUCell_calcAUC), summarized at the cell-type level.

### 2.9 Identification and metabolic activity analysis of key cells

To compare key gene expression between CKD and normal within each annotated cell type, UMAP-based visualization and the Wilcoxon rank-sum test were applied (*P* < 0.05). Cell types exhibiting significant differences in the expression of most key genes were designated key cell types for further analysis. scMetabolism (v0.2.1; https://github.com/wu-yc/scMetabolism) was used to score metabolic pathway activities in key cell types (*P* < 0.05); the top 30 pathways were visualized by ggplot2.

### 2.10 Pseudotime analysis and cellular communication analysis

Cell–cell communication among key and non-key cell types was inferred using CellChat (v1.6.1) (37) with CellChatDB.human as reference (aggregateNet; default parameters mean = trimean, trim = 0.1). Ligand–receptor interactions and signaling pathways were summarized at the cell-type level. For lineage dynamics of key cell types, neighborhood graphs were constructed (FindNeighbors, FindClusters), and Monocle (v2.30.1) (38) was used to perform pseudotime ordering and to visualize key gene expression along trajectories.

### 2.11 Quantitative real-time PCR detection of target genes in human peripheral blood samples

Peripheral blood was collected from healthy individuals (n = 16) and CKD patients (n = 20) at Guangzhou University of Traditional Chinese Medicine Shenzhen Hospital (Futian) after written informed consent. The protocol was approved by the institutional ethics committee (Approval No. GZYLL(KY)-2025-044) and conducted in accordance with the Declaration of Helsinki. Peripheral blood samples was separated by 3,000 rpm for 5 min at 4 °C and stored at −80 °C.Total RNA was extracted using Servicebio G3013 and quantified by NanoDrop. cDNA was synthesized with SweScript All-in-One RT SuperMix (One-Step gDNA Remover; Servicebio G3337). qPCR was performed with 2× Universal Blue SYBR Green Master Mix (Servicebio G3326) on a Bio-Rad CFX Connect (20 μL reactions; 95 °C denaturation; 40 cycles of 95 °C denaturation and 60 °C annealing/extension). GAPDH served as internal control, and relative expression was calculated by 2^^−ΔΔCt^. Primer sequences: METTL17 F: GCGGCACTGAAGTGTCTACTG; R: GGTCACTCCGGGTACTAAGG. SLC27A1 F: GGGGCAGTGTCTCATCTATGG; R: CCGATGTACTGAACCACCGT. GAPDH F: GGAAGCTTGTCATCAATGGAAATC; R: TGATGACCCTTTTGGCTCCC.

Clinical indices included serum creatinine (Scr), blood urea nitrogen (BUN), and 24-h proteinuria measured by standard methods. Associations between gene expression and clinical parameters were assessed by Pearson or Spearman correlation according to data distribution.

### 2.12 Animals and UUO model

Male C57BL/6 mice (8–10 weeks, 20–25 g) were obtained from Guangdong Medical Experimental Animal Center and maintained under specific pathogen-free conditions with ad libitum food and water. Unilateral ureteral obstruction (UUO) was induced under intraperitoneal pentobarbital sodium anesthesia (50 mg/kg) via midline laparotomy; the left ureter was double-ligated with 4–0 silk suture and transected between the two ligatures. Sham controls underwent identical surgery without ligation. Kidneys were harvested at day 14 for histological and molecular analyses. All procedures were approved by the IACUC (Ethics Number: 2025-1001) and complied with the NIH Guide for the Care and Use of Laboratory Animals.

### 2.13 Assessment of renal fibrosis by Masson’s trichrome staining

Kidneys were fixed in 4% paraformaldehyde, paraffin-embedded, and sectioned at 4 μm. Masson’s trichrome staining was performed per manufacturer’s instructions. Images (×200) were acquired, and five random cortical fields per animal were analyzed. Collagen-positive (blue) areas were extracted in ImageJ (Fiji) using the preset “Masson Trichrome (MT)” matrix; thresholds were applied consistently across samples, and fibrosis was quantified as collagen-positive area (%) over total tissue area.

### 2.14 Detection of target proteins in renal tissues with immunohistochemical staining

Paraffin sections were deparaffinized, rehydrated, and heat-retrieved in citrate buffer (pH 6.0). Endogenous peroxidase was blocked with 3% H_2_O_2_, followed by 5% BSA. Sections were incubated overnight at 4 °C with anti-METTL17 (1:50; Sangon D163865) and anti-SLC27A1 (1:150; ABclonal A12847), then with HRP-conjugated Rabbit Anti-Goat IgG (H+L; 1:5,000; Servicebio GB23204). DAB was used for visualization and hematoxylin for counterstaining. IHC quantification employed ImageJ with IHC Profiler, and positive area (%) was reported relative to total tissue area. (All image acquisition settings and analysis thresholds were kept constant across groups).

### 2.15 Evaluation of protein expression levels by western blot analysis

Renal tissues were lysed in RIPA buffer with protease/phosphatase inhibitors. Protein concentration was measured by BCA. Equal protein (30 μg/lane) was resolved by SDS-PAGE and transferred to PVDF. Membranes were blocked with 5% non-fat milk and incubated overnight at 4 °C with anti-METTL17 (1:500; Sangon D163865), anti-SLC27A1 (1:1,000; ABclonal A12847), and anti-GAPDH (1:4,000; Servicebio GB11002), followed by HRP-conjugated Rabbit Anti-Goat IgG (H+L; 1:10,000; Servicebio GB23204). Bands were developed by ECL and quantified in ImageJ; target proteins were normalized to GAPDH. (Protein analysis was performed in six mice per group where indicated due to atrophic UUO-14d kidneys precluding extraction).

### 2.16 Statistical analysis

Data are presented as mean ± SEM unless stated otherwise. Student’s t-test was used for two-group comparisons for normally distributed data or the Mann–Whitney U test for non-normal data. Normality was assessed as described in. Correlations were analyzed using Pearson (linear, normal data) or Spearman (rank-based, non-parametric). Analyses were conducted in GraphPad Prism 9.0; *P* < 0.05 was considered statistically significant. (Two-tailed tests were used throughout).

## 3 Results

### 3.1 Identification of 67 candidate genes

**Figure 1:**
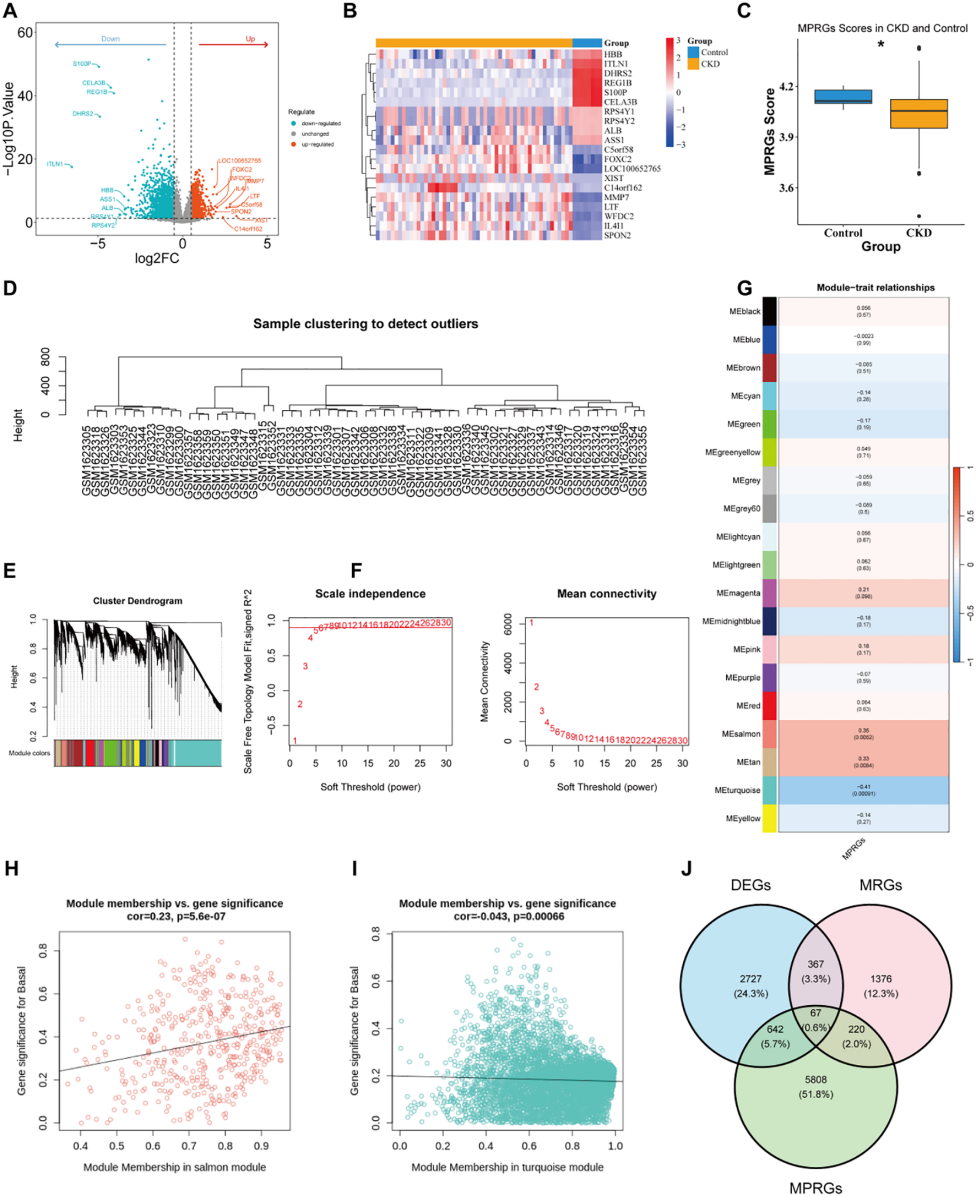
Identification of candidate genes in CKD through integrative transcriptomic analysis. **(a, b)** Volcano plot **(a)** and expression heat map **(b)** of DEGs in CKD and control groups. **(c)** Differences in MPRGs scores between the CKD group and the control group. **(d)** Sample clustering tree of GSE66494 samples. **(e)** Soft-threshold scale independence and mean connectivity map. **(f)** Hierarchical clustering tree, with 18 modules in total. **(g)** Module and trait correlation diagram. **(h, i)** Scatter plots of GS and MM association strengths for key modules (left: MEsalmon; right: MEturquoise). **(j)** Venn diagram showing the overlap among DEGs, MPRGs, and MRGs.

Findings of the differential expression analysis revealed that as compared to the control group, there were a total of 3,803 DEGs in the CKD group, among which 1,705 were upregulated and 2,098 were downregulated (**Figures 1a, b**). There was a significant difference in the MPRG score between the CKD group and the control group (*P* = 0.048), indicating that MPRG may be associated with this disease (**Figure 1c**). No outlier samples were detected by WGCNA (**Figure 1d**), and the optimal soft-threshold was 7 (**Figure 1e**). A total of 18 gene modules were obtained by WGCNA (**Figure 1f**). Subsequently, two positive and negative modules most related to the MPRGs score phenotype were selected, MEsalmon (cor = 0.35, *p* = 0.0052), including 463 genes, and MEturquoise (cor = -0.41, *p* = 0.0009), including 6,274 genes (**Figure 1g**). These two modules included 6,737 module genes. The association relationships between GS and MM of the two module genes were positive (**Figures 1h, i**). Finally, a total of 67 candidate genes were identified through the intersection of DEGs, MRGs, and module genes (**Figure 1j**) (**Supplementary Table S2**).

### 3.2 Enrichment and PPI network analysis in 67 candidate genes

**Figure 2:**
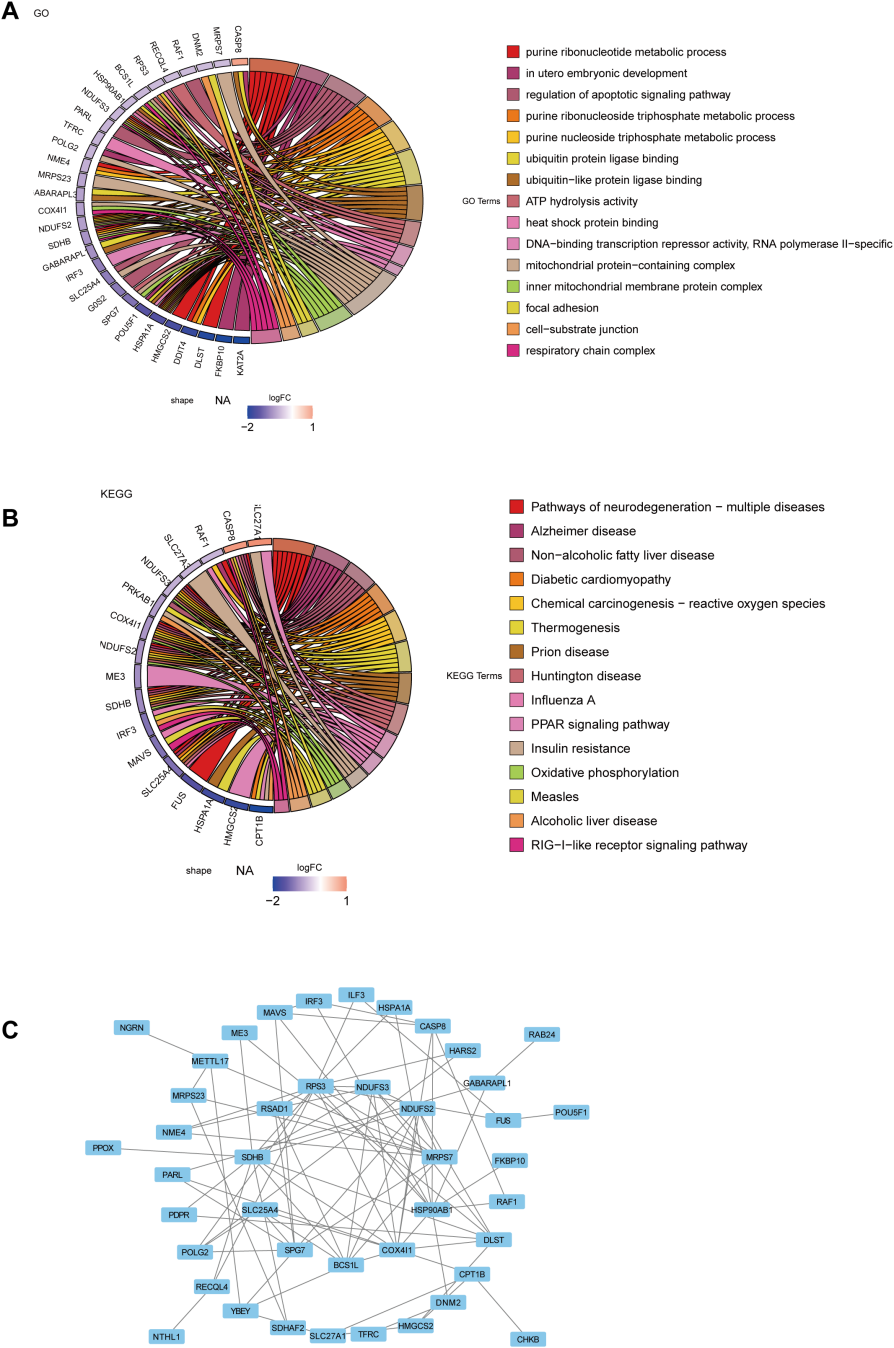
Functional enrichment and protein-PPI analysis of candidate genes in CKD. **(a)** GO enrichment analysis of candidate genes. **(b)** KEGG pathway enrichment analysis. **(c)** PPI network construction.

A total of 67 candidate genes were enriched in 381 biological functions and pathways, with 294 BPs, 27 CCs, and 60 MFs being significantly enriched (*p*.adjust < 0.05) (**Supplementary Table S3**). The first 15 GO entries enriched contain the following processes: purine ribonucleotide metabolic process, *in utero* embryonic development, regulation of apoptotic signaling pathway, purine ribonucleoside triphosphate metabolic process (**Figure 2a**). The first 15 pathways enriched in KEGG pathway include Pathways of neurodegeneration-multiple diseases, Non-alcoholic fatty liver disease, Diabetic cardiomyopathy, Chemical carcinogenesis-reactive oxygen species, and Thermogenesis, and so on (**Figure 2b**; **Supplementary Table S4**). After that, a total of 43 nodes and 89 sides were shown in a PPI network diagram (No interactions was found for the proteins corresponding to the 24 genes) (**Figure 2c**). HSP90AB1, NDUFS2, MRPS7, and RPS3 were more abundantly associated with other genes.

### 3.3 Identification and validation of 2 key genes

**Figure 3:**
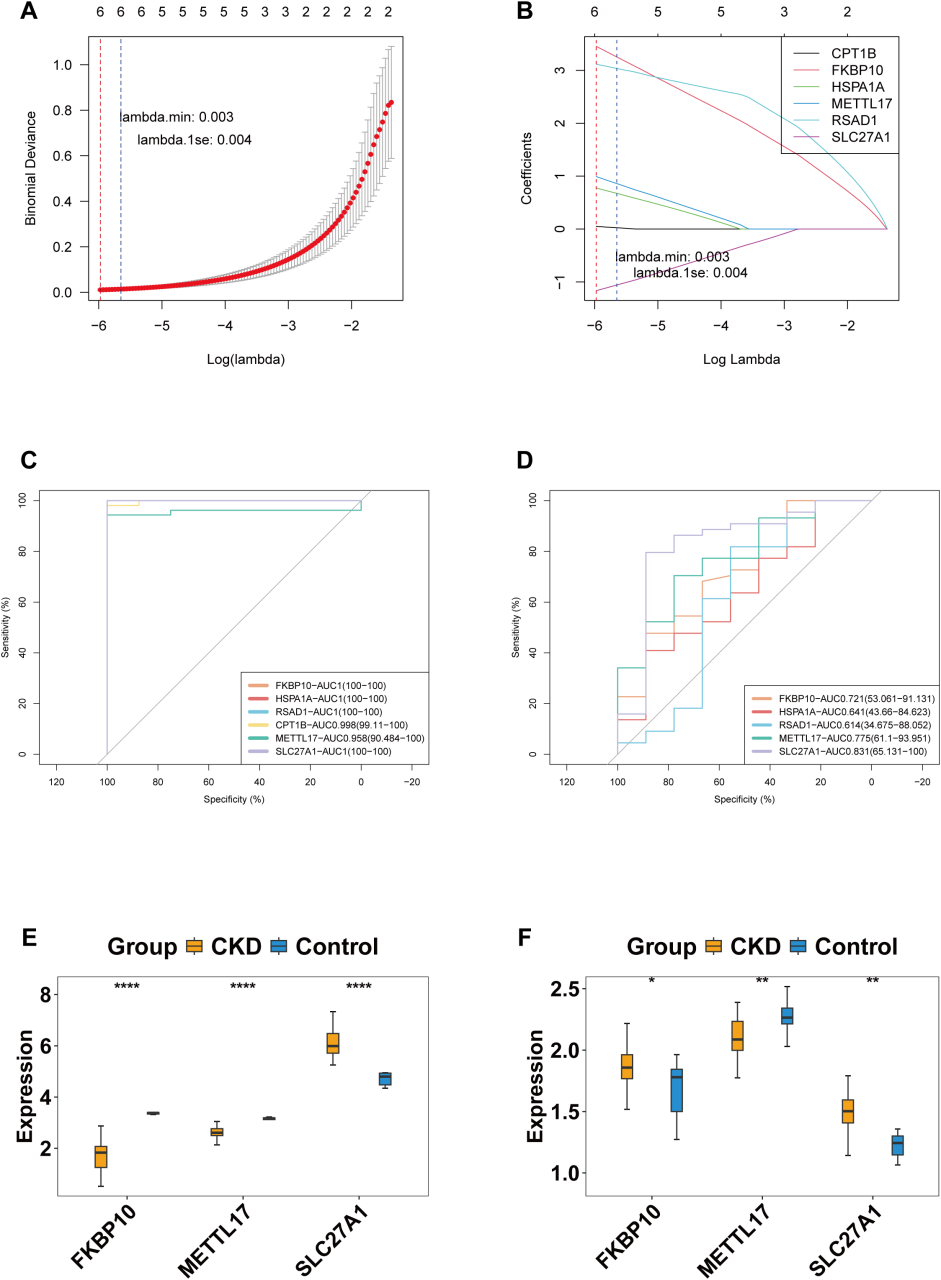
Identification and validation of key genes in CKD. **(a, b)** LASSO regression analysis in the GSE66494 dataset identified eight candidate genes associated with CKD. **(c-d)** ROC curve analysis of characteristic genes in the training set **(c)** and validation set **(d)**. **(e, f)** Expression levels of candidate key genes in the training set **(e)** and validation set **(f)**. P < 0.05 (*), P < 0.01 (**), P < 0.001 (***).

LASSO regression in GSE66494 selected eight signature genes (**Figures 3a, b**). ROC curve analysis showed that six of these had AUC > 0.7 in both GSE66494 and GSE180394 (**Figures 3c, d**). Among them, two genes (METTL17 and SLC27A1) exhibited consistent expression patterns (upregulated in CKD with *P* < 0.05) in both datasets (**Figures 3e, f**). These two genes were defined as “key genes” for further analyses.

### 3.4 GSEA and chromosomal localization of METTL17 and SLC27A1

**Figure 4:**
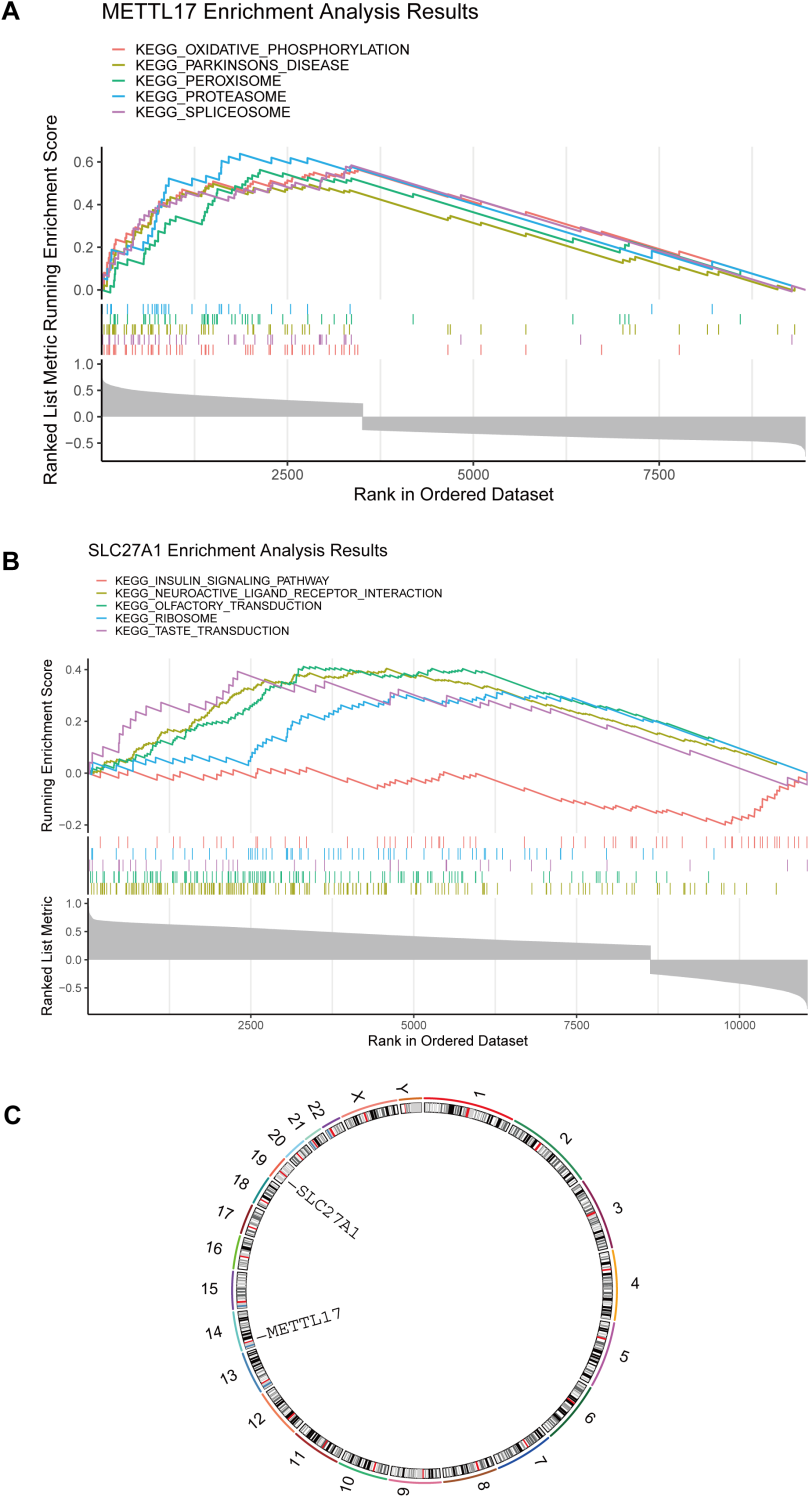
Functional enrichment and chromosomal localization of METTL17 and SLC27A1. **(a)** GSEA (METTL17) gene set enrichment analysis. **(b)** GSEA (SLC27A1) gene set enrichment analysis diagram. **(c)** Chromosomal location of key genes (METTL17, SLC27A1).

GSEA of genes correlated with METTL17 revealed enrichment in ribosome, RNA transport, and oxidative phosphorylation pathways (**Figures 4a, b**). For SLC27A1, enriched pathways included fatty acid metabolism, PPAR signaling, and peroxisome functions (**Figures 4c, d**). Chromosomal mapping placed METTL17 on chromosome 11 and SLC27A1 on chromosome 19 (**Figure 4e**).

### 3.5 Analysis of key genes with differential immunity cells

**Figure 5:**
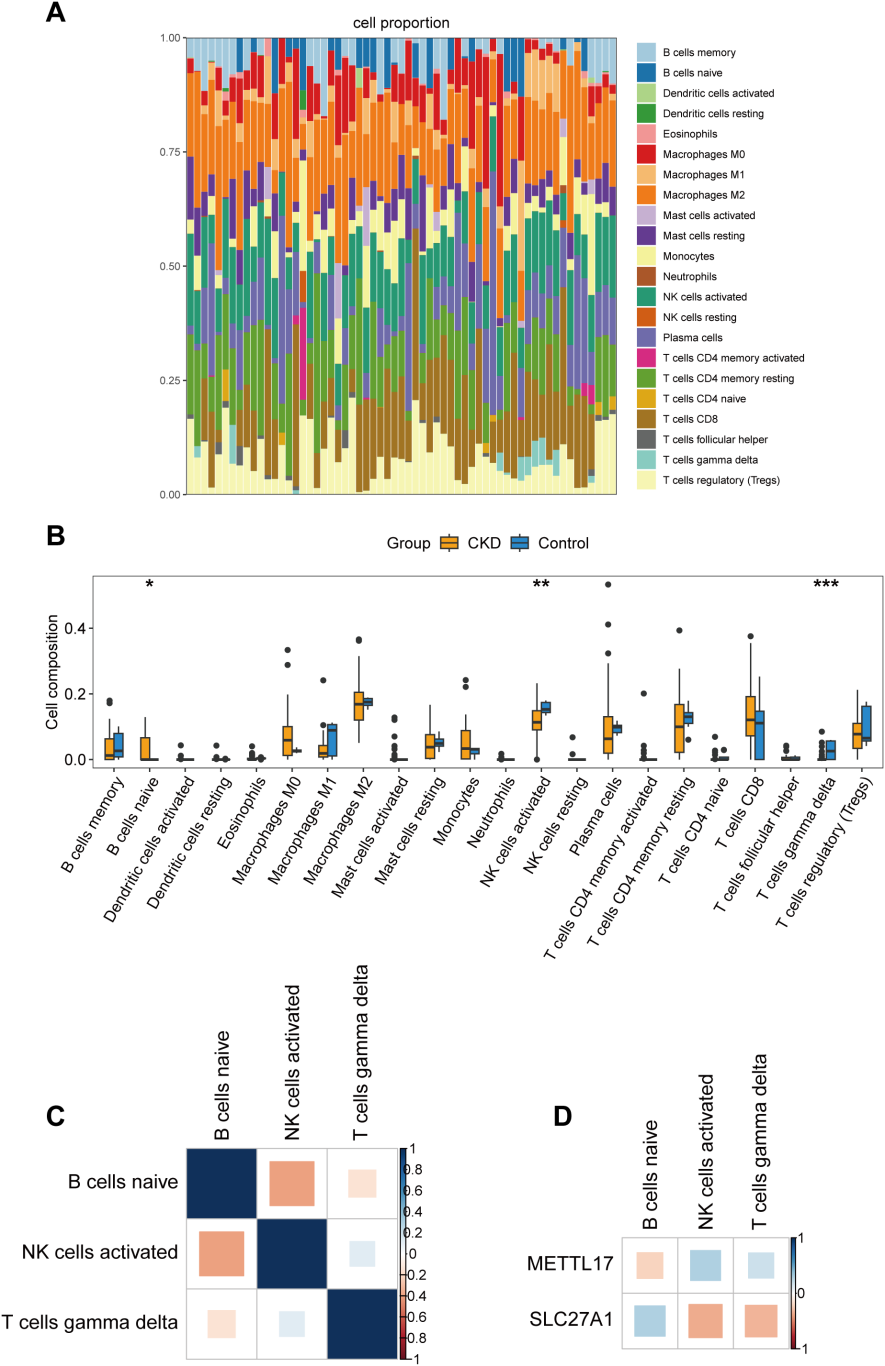
Immune infiltration analysis in CKD. **(a)** Abundance of immune cell infiltration in the CKD group and the control group. **(b)** Correlation heatmap of differential immune cells. **(c)** Analysis of differences in immune cell infiltration between the CKD group and the control group. **(d)** Correlation analysis between differential immune cells and key genes (METTL17 and SLC27A1). P < 0.05 (*), P < 0.01 (**), P < 0.001 (***).

In the GSE66494 dataset, 41 samples were retained for subsequent analysis (*P* < 0.05), with the distribution proportions of 22 types of immune cells varied among different samples (**Figure 5a**; **Supplementary Figure S1**). According to the results of the Wilcoxon test, a total of 3 types of differentially distributed immune cells were obtained: naive B cells, activated NK cells, and gamma delta T cells (*P* < 0.05) (**Figure 5b**). Additionally, the correlation analysis indicated that activated NK cells were significantly negatively correlated with naive B cells (cor < -0.3, *P* < 0.005) (**Figure 5c**). METTL17 was positively correlated with naive B cells (cor > 0.3, *P* < 0.005) and was strongly inversely correlated with activated NK cells (cor < -0.3, *P* < 0.005). SLC27A1 was significantly negatively associated with naive B cells (cor < -0.3, *P* < 0.005) and significantly positively associated with activated NK cells (cor > 0.3, *P* < 0.005) (**Figure 5d**).

### 3.6 Network construction related to METTL17 and SLC27A1

**Figure 6:**
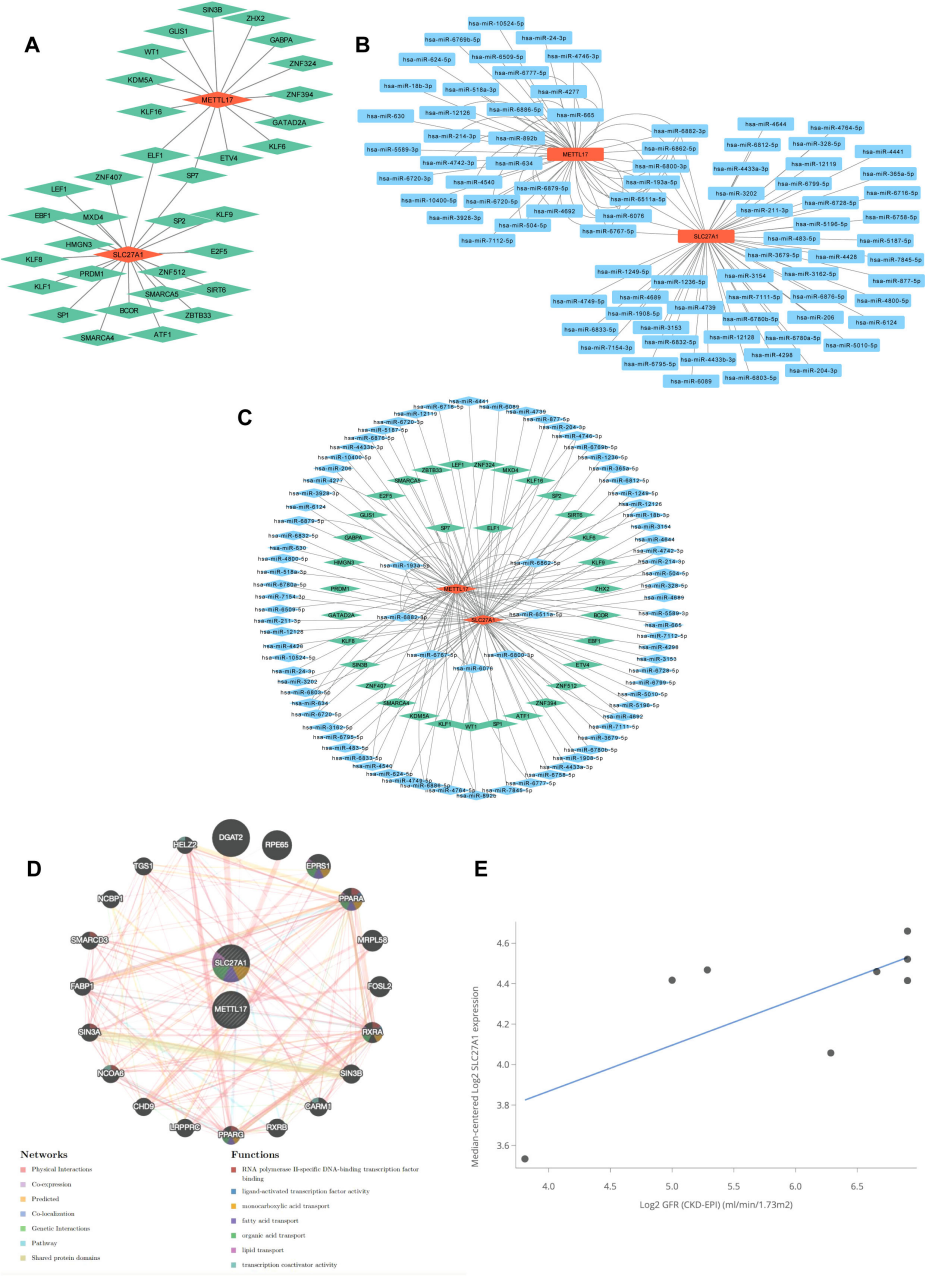
Construction of regulatory and functional networks for METTL17 and SLC27A1. **(a)** TF-mRNA network. **(b)** mRNA-miRNA network. **(c)** TF-mRNA-miRNA regulatory network. **(d)** GeneMANIA network construction. **(e)** Clinical correlation analysis revealing a significant positive association between SLC27A1 expression and renal function indices (serum creatinine; Pearson r = 0.730, *P* = 0.04).

In an attempt to uncover the molecular regulatory mechanisms related to METTL17 and SLC27A1, in the TF-key gene network, a total of 33 transcription factors (TFs) regulate the 2 key genes. Among them, ELF1 and SP7 jointly regulate the two key genes (**Figure 6a**). In the miRNA-key gene network, 83 microRNAs (miRNAs) were predicted for the two key genes. There are a total of 117 relationships between these 83 miRNAs and the two key genes. Hsa-miR-214-3p, hsa-miR-483-5p, and hsa-miR-193a-5p have closer associations with the two key genes (**Figure 6b**). In the TF-miRNA-key gene network, there are 152 regulatory relationships among the 33 TFs, 83 miRNAs, and the two key genes, indicating a relatively tight regulatory relationship among the three (**Figure 6c**). The GeneMANIA network showed that the 2 key genes were predicted to be related to 20 other genes and 243 correlations between these 22 genes. Associations mainly existed in aspects such as physical interactions and co-expression. These genes were associated with ligand-activated transcription factor activity, fatty acid transport, organic acid transport, lipid transport, and transcription activator activity, each having corresponding functions (**Figure 6d**). In terms of clinical relevance, SLC27A1 expression was significantly correlated with key renal function parameters (cor=0.730, *P* = 0.04) (**Figure 6e**).

### 3.7 Single-cell sequencing reveals proximal tubular cells and smooth muscle cells as key cell populations in CKD

**Figure 7:**
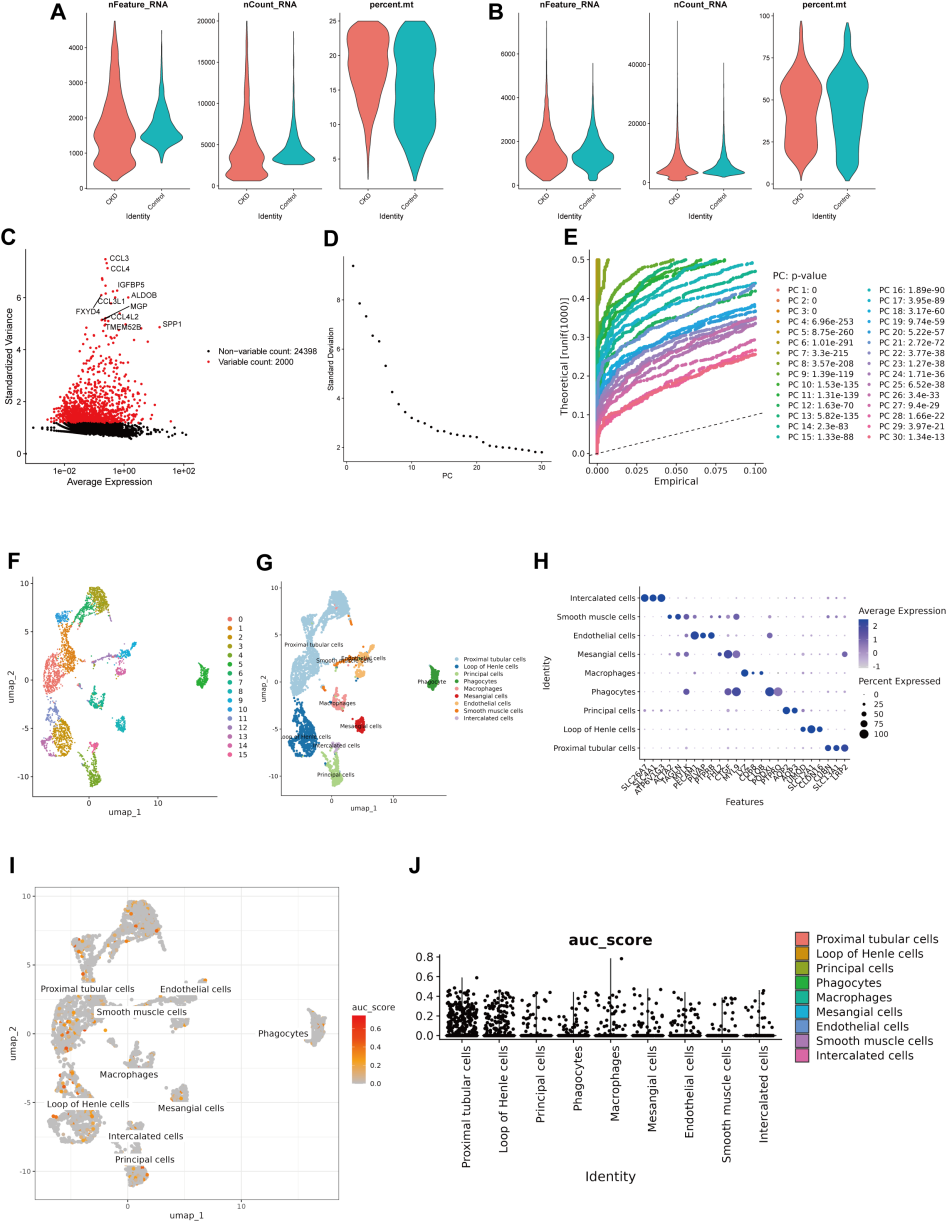
Single-cell transcriptomic landscape of CKD kidneys. **(a, b)** Quality control plots of nFeature_RNA, nCount_RNA, and percent.mt of the GSE171314 dataset. **(c)** Screening of highly variable genes. **(d)** and PCA principal component analysis diagram. **(e, f)** UMAP visualization of single-cell clustering based on the top 30 PCs, revealing 16 distinct clusters. **(g, h)** Annotation of cell clusters into nine major renal cell types. **(i, j)** Scatter plot of AUCell scores of key genes **(i)** and AUCell scores of key genes in various cell types **(j)**.

After preliminary screening, a total of 26,398 genes were obtained from the GSE171314 dataset (**Figures 7a, b**). After normalizing and reducing the dimensionality of the data, 2,000 highly variable genes, 30 PCs, and 16 cell clusters were obtained (**Figures 7c-f**). A total of 16 cell clusters were annotated into 9 cell types, including Mesangial cells, Endothelial cells, Phagocytes, Proximal tubular cells (PTCs), Loop of Henle cells, Principal cells, Smooth muscle cells (SMCs), Intercalated cells, and Macrophages (**Figures 7g-h**; **Supplementary Figure S2**). Subsequently, according to the AUcell analysis, the AUCell scores of two key genes in Proximal tubular cells were the highest, followed by those in Loop of Henle cells (**Figures 7i, j**).

**Figure 8:**
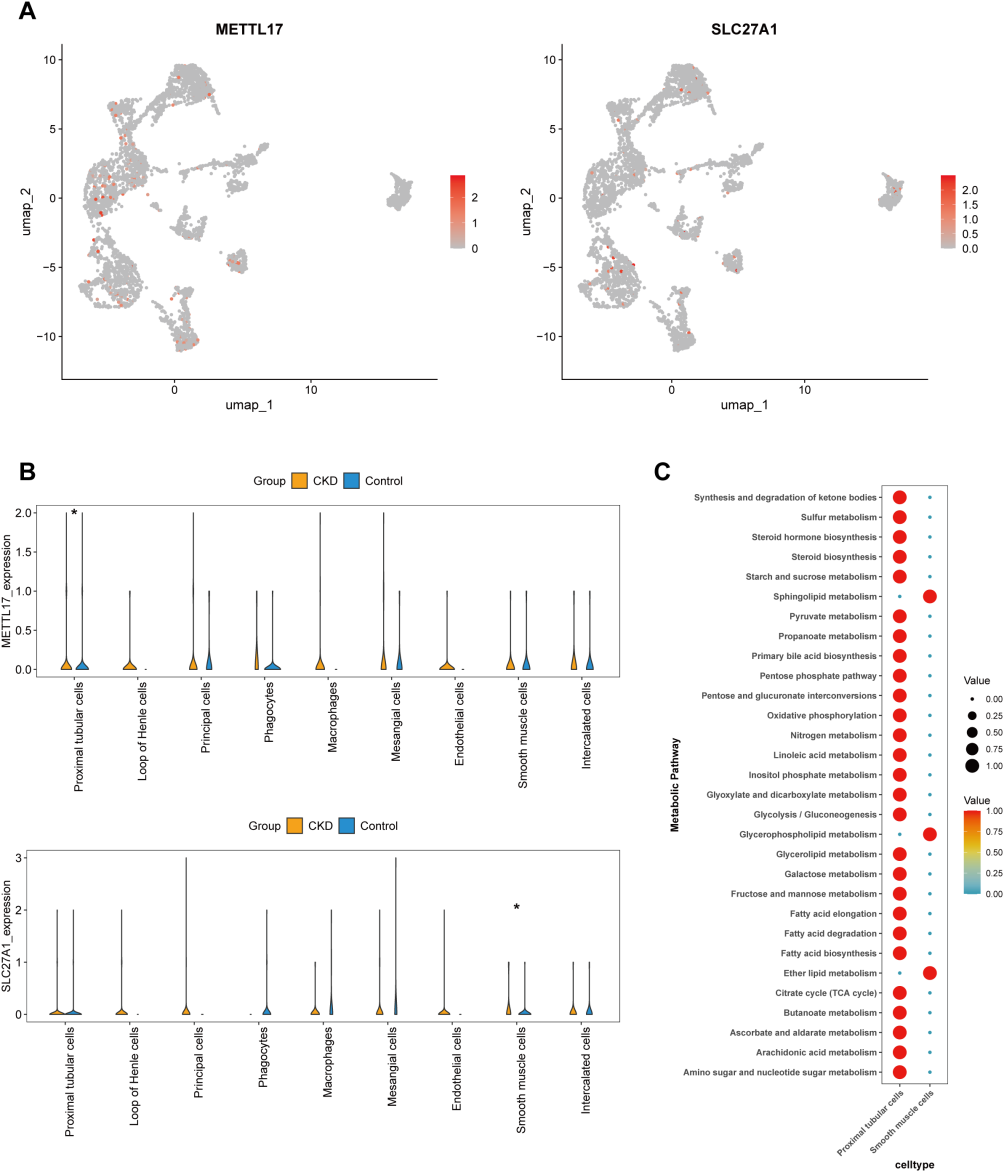
Identification of PTCs and SMCs as key cell populations in CKD. **(a, b)** Expression of key genes in various cell types. **(c)** Analysis of pathway activity in key cell populations. P < 0.05 (*), P < 0.01 (**), P < 0.001 (***).

In addition, according to the UMAP analysis and Wilcoxon test, SLC27A1 and METTL17 displayed marked differences (*P* < 0.05) in PTCs and SMCs between the CKD group and the normal group. Therefore, these two types of cells were identified as key cells in CKD (**Figures 8a, b**). PTCs had high scores in 27 pathways, while SMCs had high scores in three pathways: Sphingolipid metabolism, Glycerophospholipid metabolism, and Ether lipid metabolism (**Figure 8c**).

### 3.8 Cell communication of key cells and pseudotime analysis

**Figure 9:**
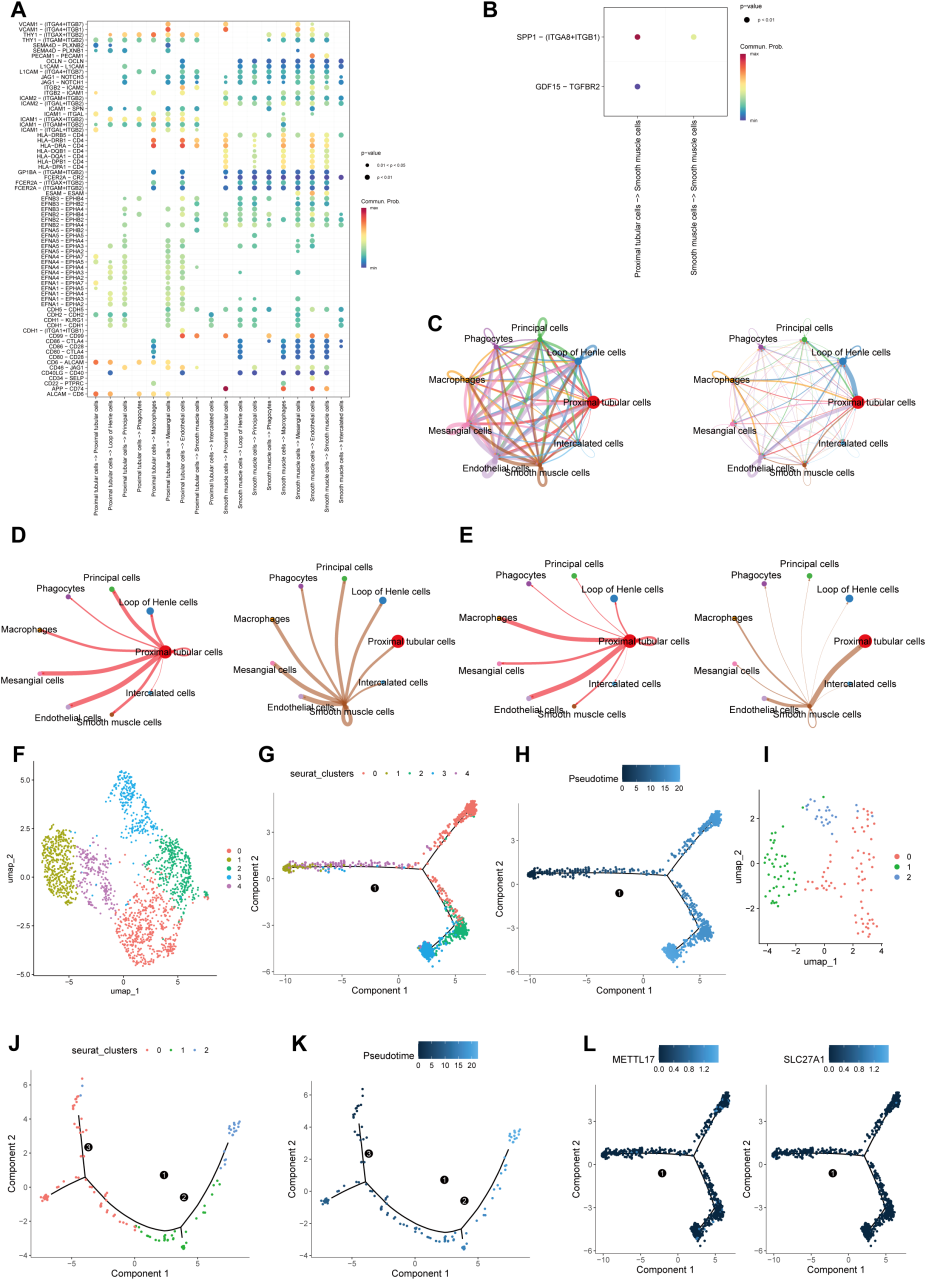
Cell-cell communication and pseudotime trajectory analyses of PTCs and SMCs in CKD. **(a)** Cell-cell interaction network. **(b)** Key ligand-receptor signaling relationships enriched in CKD. **(c)** Strength of intercellular communication among annotated renal cell types, showing enhanced signaling between PTCs and loop of Henle cells, endothelial cells, and macrophages. **(d, e)** Key cell-cell interaction strength. **(f)** UMAP clustering of PTCs into five subgroups. **(g, h)** Pseudo-temporal analysis of proximal tubular cells. **(i)** UMAP clustering of SMCs into three subgroups. **(j, k)** Pseudotime analysis of SMCs showing Subgroup 0 in the early stage, Subgroup 1 in the transitional stage, and Subgroup 2 in the late stage. **(l)** Differentiation trajectories of key genes in proximal tubular cells.

Additionally, cell-communication analysis revealed the interactions between PTCs and SMCs and other annotated cell types. The analysis results showed that, in both the CKD group and the normal group, PTCs regulated the signals between key cells and other receptors through the high expression levels of HLA-CD4 and CD6-ALCAM, as well as the low expression of EFNA1, EFNA4, and EFNA5. For SMCs, they mostly achieved intercellular signal transmission and exerted functional influence by the low expression of PCER2A, L1CAM, JAG1, CD80, CD86 and their ligands, along with the high expression of HLA-CD4 and APP-CD74. In the communication relationship between SMCs and PTCs, the high-expression state of the APP-CD74 ligand-receptor pair played a crucial role (**Figure 9a**), and this communication mainly relied on the relationships of SPP1-(ITGAB + ITGB1) and GDF15-TGFBR2 (**Figure 9b**). PTCs demonstrated relatively strong inter-cellular communication capabilities with Loop of Henle Cells and Endothelial Cells (**Figure 9c**). Specifically, PTCs had a relatively strong communication ability with Endothelial Cells and Macrophages, while SMCs had a relatively strong communication with PTCs (**Figures 9d, e**).

This study indicated that, after dimensionality-reduction clustering, PTCs were divided into five subgroups (**Figure 9f**). Subgroup 1 and Subgroup 4 were in the early stage, while Subgroup 0, Subgroup 2, and Subgroup 3 were in the end-stage (Subgroup 2 and Subgroup 3 were on the same branch) (**Figures 9g, h**). SMCs were divided into three subgroups (**Figure 9i**), among which Subgroup 0 was in the early stage, Subgroup 1 was in the transitional stage, and Subgroup 2 was in the end-stage (**Figures 9j, k**). Notably, in PTCs, the key genes were located in the end-stage (**Figure 9l**).

### 3.9 Clinical validation of METTL17 and SLC27A1 in CKD patients

**Figure 10:**
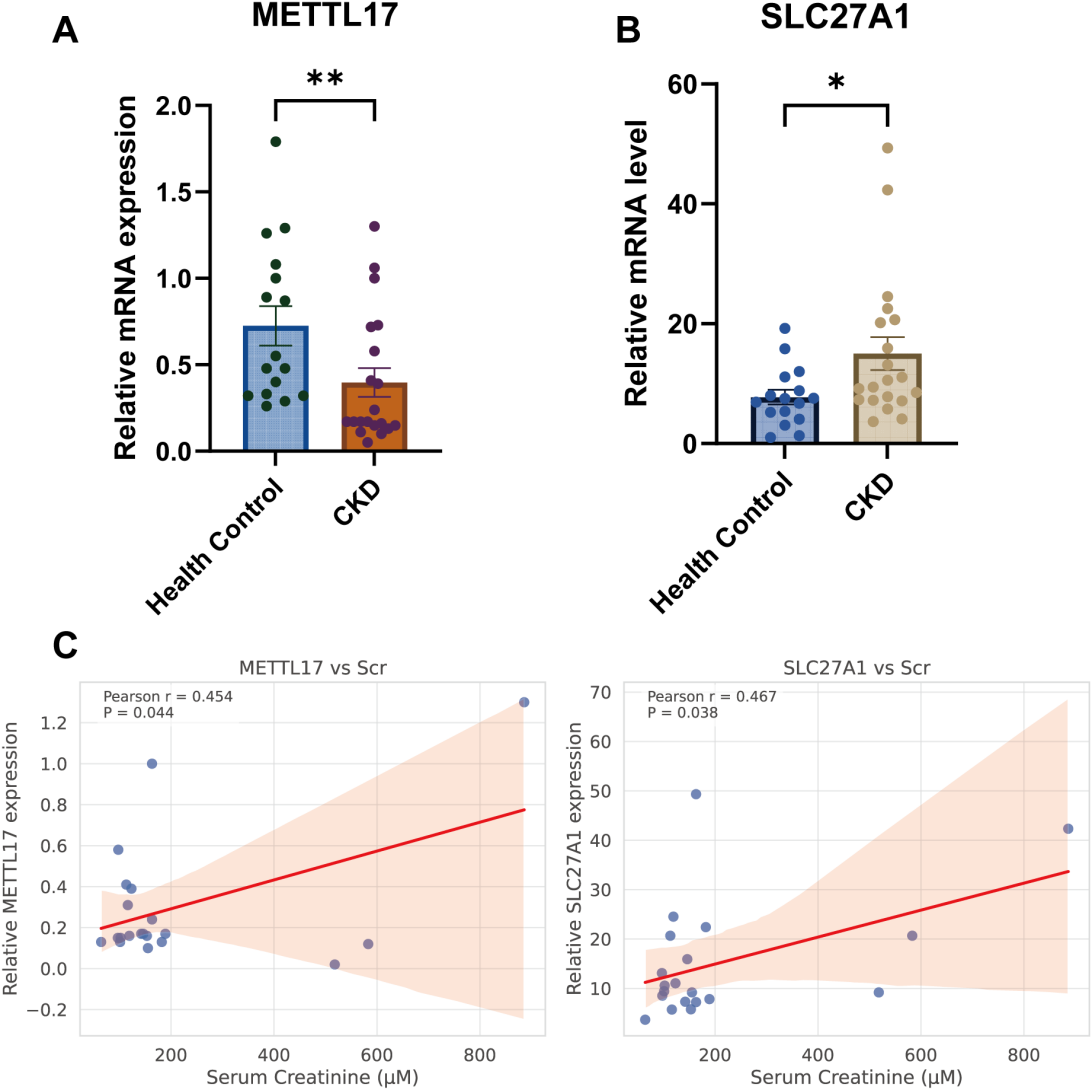
Clinical validation of METTL17 and SLC27A1 in CKD patients. **(a, b)** Comparison of SLC27A1 and METTL17 mRNA levels in peripheral blood samples between healthy controls (n=16) and patients with chronic kidney disease (n=20). **(c)** Correlation between peripheral blood samples METTL17 and SLC27A1 mRNA expression and serum creatinine in CKD patients (n=20). P < 0.05 (*), P < 0.01 (**).

To validate the findings from bioinformatics analyses, peripheral blood samples levels of METTL17 and SLC27A1 mRNA were quantified in 20 CKD patients and 16 healthy controls by qPCR. Baseline demographic and clinical characteristics of participants are summarized in **Table 1**. Consistent with the *in silico* predictions, METTL17 expression was significantly decreased in CKD patients compared with controls (*P* < 0.01), whereas SLC27A1 expression was markedly increased (*P* < 0.01) (**Figures 10a, b**).

**Table 1:** Clinical and demographic features of patients with CKD and controls.

Feature	Healthy controls	CKD
SEX	F7/M9	F13/F7
Age(years)	46.5±13.47	66.5±10.26
Serum creatinine (uM)	59.5±14.51	144±296.09
Serum blood urea nitrogen (uM)	5.035±1.14	8.85±10.90
Serum uric acid (uM)	348±76.46	381±111.43
24-h proteinuria (g)	n.d.	0.42±2.17
CKD I	n.a.	1
CKD II	n.a.	2
CKD III	n.a.	9
CKD IV	n.a.	3
CKD V	n.a.	5

Correlation analyses were further performed to assess the relationship between gene expression and renal function indices in CKD patients. METTL17 showed a modest positive correlation with serum creatinine (Pearson r = 0.454, *P* = 0.044; **Figure 10c**). However, this association was not robust: it became non-significant after excluding extreme creatinine values (>300 μM; r = 0.067, *P* = 0.800) or after winsorization at 300 μM (r = 0.236, *P* = 0.317). Importantly, Spearman’s rank test, which does not assume normality, did not confirm this association, indicating that the correlation may be driven by linear trends influenced by extreme values rather than a robust monotonic relationship. In contrast, SLC27A1 expression was positively correlated with serum creatinine (Pearson r = 0.467, *P* = 0.038; **Figure 10c**) and showed a trend toward correlation with blood urea nitrogen (Pearson r = 0.390, *P* = 0.089), while no significant association was found with 24-hour proteinuria (*P* > 0.05). Spearman’s analysis yielded similar non-significant trends, further reflecting the modest sample size and the heterogeneity of CKD patients.

### 3.10 *In vivo* validation of METTL17 and SLC27A1 in a UUO mouse model of renal fibrosis

**Figure 11:**
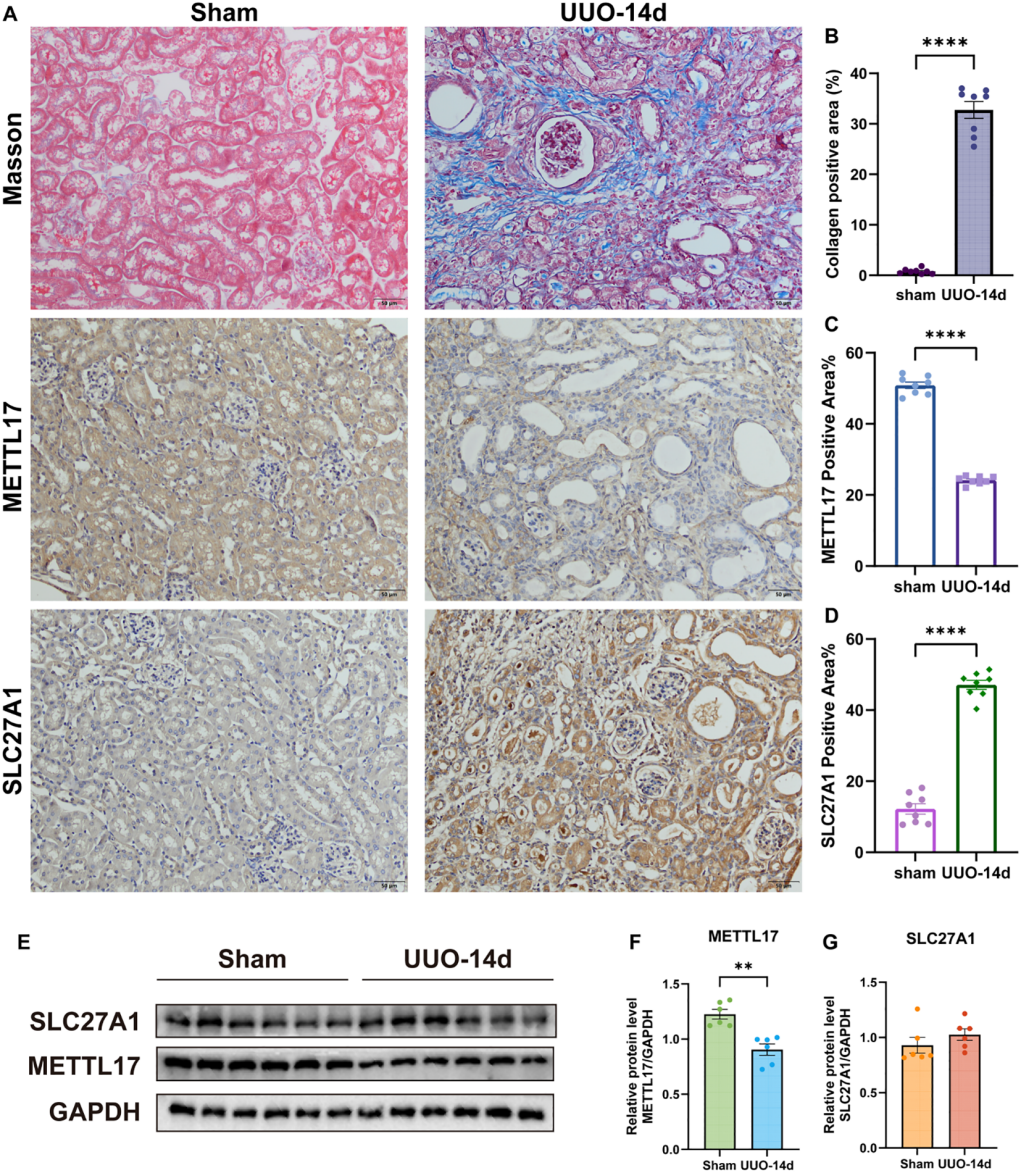
*In vivo* validation of METTL17 and SLC27A1 in a UUO mouse model of renal fibrosis. **(a)** Representative immunohistochemical staining of METTL17 and SLC27A1 in kidney sections from sham-operated and UUO-14d mice. **(b)** Masson’s trichrome staining confirmed the presence of collagen deposition after UUO. **(c, d)** Quantification of positive staining areas, showing decreased METTL17 and increased SLC27A1 expression in UUO kidneys. **(e)** Representative Western blot bands of METTL17 and SLC27A1 in sham and UUO kidneys. **(f, g)** Density quantitative analysis results of Western blot. P < 0.01 (**), P < 0.0001 (****).

The success of the UUO model was confirmed by histological analysis. Masson’s trichrome staining demonstrated markedly increased collagen deposition in UUO kidneys compared with sham-operated controls (*P* < 0.01; **Figures 11a, b**), indicating successful induction of renal fibrosis. Immunohistochemical analysis showed that METTL17 expression was significantly reduced in renal tubular epithelial cells of UUO mice (*P* < 0.01; **Figures 11a, c**), whereas SLC27A1 expression was significantly increased (*P* < 0.05; **Figures 11a, d**). These changes were consistent with the expression patterns observed in CKD patients. Western blotting further validated these findings, showing that METTL17 protein levels were significantly downregulated in UUO kidneys compared with sham controls (*P* < 0.05), while SLC27A1 protein levels showed an upward trend without reaching statistical significance (ns; **Figures 11e-g**). Protein analysis was performed in six mice per group, as twokidneys from UUO mice at day 14 were too atrophic for protein extraction, which may have contributed to the limited statistical power.

## 4 Discussion

The global burden of CKD is steadily rising, and the World Health Organization has designated CKD as a global health priority (4). Macrophage polarization represents a defining feature of CKD progression (39). M1 macrophages release pro-inflammatory cytokines that impair renal function and promote fibrosis (40), whereas M2 macrophages secrete anti-inflammatory cytokines that facilitate renal repair (41). Emerging evidence indicates that disruption of mitochondrial homeostasis, altered bioenergetics, and organelle stress are critical contributors to kidney disease (42). Mitochondrial dysfunction results in reduced ATP generation and elevated reactive oxygen species (ROS) production, which collectively exacerbate renal impairment (43, 44). Understanding the underlying mechanisms of CKD and identifying novel biomarkers or therapeutic targets remain critical. Through cross-dataset analyses and validations in peripheral blood samples from CKD patients and in UUO mouse models, this study identified two key genes-METTL17 and SLC27A1-that exhibit consistently dysregulated expression in CKD and show robust diagnostic performance (AUC > 0.75) across multiple datasets, suggesting that these genes may serve as important regulators of CKD progression.

Methyltransferase like 17 (METTL17) belongs to the methyltransferase-like protein family and is localized to mitochondria (45). It regulates mitochondrial gene expression through rRNA methylation and maintenance of oxidative phosphorylation (OXPHOS) (46). In this study, METTL17 was found to be consistently downregulated in CKD samples and functionally associated with the OXPHOS pathway, which has been implicated in driving vascular calcification in CKD (47). METTL17 is predominantly expressed in PTCs-the most metabolically active cells in the nephron-which rely heavily on mitochondrial OXPHOS to sustain ATP-dependent solute reabsorption (48). Downregulation of METTL17 may impair mitochondrial transcription and translation, resulting in reduced OXPHOS efficiency and excessive accumulation of reactive oxygen species, ultimately contributing to renal tubular injury, fibrosis, and vascular calcification. Furthermore, given the close association between oxidative phosphorylation and macrophage polarization toward the M2 phenotype (49), and evidence indicating that METTL17 suppresses inflammatory responses and M1 macrophage polarization through STAT1 RNA methylation-whereas its knockdown promotes M1 polarization (50)-we hypothesize that reduced METTL17 expression may exacerbate CKD progression by reshaping the immune microenvironment. This study broadens current understanding of the role of methyltransferases in disease pathogenesis. Beyond its established role in rRNA methylation, METTL17 may function as a novel non-histone methylation regulator, analogous to protein arginine methyltransferases (PRMTs) and lysine methyltransferases (KMTs), which are known to modulate pathological processes-such as tumorigenesis and immune responses-by regulating the activity, interactions, and stability of target proteins (51). In conclusion, METTL17 downregulation may promote CKD progression through a dual mechanism involving impaired renal tubular energy metabolism and enhanced macrophage M1 polarization. However, further studies are required to validate the effects of METTL17 on mitochondrial function and fibrosis in cellular models and to elucidate its role in macrophage polarization and CKD progression using genetic knockout approaches.

Solute carrier family 27 member 1 (SLC27A1) facilitates long-chain fatty acid uptake and plays a central role in lipid homeostasis (52). In these tissues, SLC27A1 facilitates cellular energy supply by mediating the uptake of fatty acids (FAs) (53). In CKD, SLC27A1 was markedly upregulated and positively correlated with serum creatinine levels, suggesting a link to renal functional decline (54). Aberrant SLC27A1 expression may exacerbate CKD progression by (i) increasing fatty acid flux into tubular and vascular cells, (ii) enhancing lipid peroxidation and ROS generation, and (iii) triggering pro-inflammatory cascades (55). Inflammation is a well-established driver of CKD progression (56), and aberrant SLC27A1 expression may contribute to CKD onset and progression by modulating inflammation- and oxidative stress-related signaling pathways. Studies have shown that activated NK cells can further promote macrophage polarization toward the M1 phenotype and accelerate fibrotic progression by stimulating the release of pro-inflammatory cytokines, such as interferon-γ (57). These findings suggest that during CKD progression, upregulation of SLC27A1 may exacerbate lipid metabolic disturbances and consequently initiate a cascade of pathological processes, including inflammation, mitochondrial dysfunction, and oxidative stress. These processes not only directly induce renal tubular injury and vascular remodeling but also synergistically promote CKD onset and progression by disrupting renal energy homeostasis and immune cell function. However, the precise mechanisms and their contributions to CKD pathogenesis require further experimental validation.

The findings of this study suggest that METTL17 and SLC27A1 converge at the intersection of mitochondrial dysfunction and immune imbalance in CKD, constituting a potential pathogenic axis. On the one hand, downregulation of METTL17 in PTCs may impair mitochondrial oxidative phosphorylation (OXPHOS), resulting in compromised cellular energy metabolism and excessive reactive oxygen species (ROS) production (58). This mitochondrial dysfunction may not only promote renal tubular injury but also establish a pro-inflammatory microenvironment that favors macrophage polarization toward the M1 phenotype (59, 60). In addition, the loss of METTL17-associated anti-inflammatory RNA methylation activity may further amplify this polarization process (61). On the other hand, upregulation of SLC27A1 may disrupt lipid homeostasis, potentially inducing mitochondrial β-oxidation overload and lipid peroxidation, thereby exacerbating mitochondrial stress and ROS generation (62). The resulting lipotoxic and oxidative stress signals may enhance pro-inflammatory cytokine release through activation of innate immune cells, such as NK cells, thereby further promoting macrophage M1 polarization (63). Consequently, reduced METTL17 expression and elevated SLC27A1 expression may synergistically disrupt mitochondrial–immune homeostasis: impaired mitochondria fail to meet cellular metabolic demands and generate stress signals, while dysregulated lipid metabolism and the ensuing inflammatory responses drive macrophage polarization toward a pro-fibrotic M1 phenotype (64). This self-reinforcing cycle of metabolic impairment, oxidative stress, and inflammatory amplification may ultimately accelerate renal tubular injury, interstitial fibrosis, and vascular lesions—hallmark features of CKD progression (65).

Single-cell analysis revealed that PTCs and SMCs are central hubs of gene dysregulation. Ligand–receptor analysis identified APP–CD74 and SPP1–ITGAB/ITGB1 as dominant communication axes, suggesting that metabolic dysfunction in PTCs and SMCs also reshapes intercellular signaling. Fan et al. reported that RTN3 deficiency activates the IGF2–JAK2 signaling pathway in proximal tubular epithelial cells (PTECs), thereby promoting CKD onset and renal fibrosis (66). In hypoxic renal injury, small extracellular vesicles derived from human primary PTECs mediate synchronized tubular ferroptosis, thereby facilitating the transition from acute kidney injury (AKI) to CKD. Vascular calcification (VC), a common complication in CKD patients, is strongly associated with adverse cardiovascular outcomes (67). Under CKD-associated pathological conditions, vascular SMCs can undergo osteogenic transdifferentiation, resulting in extracellular calcium phosphate deposition within the vascular wall and promoting VC development (68, 69). Collectively, dysfunction of PTCs and SMCs may drive CKD progression by contributing to renal fibrosis and vascular calcification.

In summary, these findings suggest that METTL17 and SLC27A1 may contribute to CKD pathogenesis through distinct mechanisms, with downregulated METTL17 potentially impairing protective metabolic functions in tubular cells, and upregulated SLC27A1 promoting lipid metabolic dysregulation. The integration of patient-derived and experimental model data reinforces the translational relevance of these genes and supports their candidacy as biomarkers or therapeutic targets for renal fibrosis.

However, this study has several limitations. First, the clinical relevance analysis is relatively fragile because of the limited sample size and the inability to fully adjust for potential confounders, necessitating further validation in larger, independent cohorts. At the protein level, Western blot analysis confirmed a significant downregulation of METTL17, whereas SLC27A1 exhibited an upward trend that did not reach statistical significance, likely reflecting reduced statistical power arising from the limited sample size. Furthermore, the results are highly dependent on data quality and algorithmic performance; given the complexity and heterogeneity of biological systems, the current analyses may not fully capture the underlying biological processes. In addition, current experimental validation primarily focuses on gene and protein expression and has not yet established the causal links between METTL17 or SLC27A1 and mitochondrial function or macrophage polarization through genetic perturbation experiments. Future studies should expand sample sizes and systematically evaluate the direct effects of these genes on mitochondrial metabolic activity, membrane potential, and macrophage phenotypic shifts by generating overexpression or knockdown cell models, thereby clarifying their roles in CKD pathogenesis. Moreover, further investigation is required to delineate the regulatory relationship between METTL17 and SLC27A1, for example by assessing the expression of one gene following silencing or overexpression of the other, to clarify their hierarchical positions within the molecular network. Finally, *in vitro* co-culture systems will be needed to elucidate the mechanisms by which immune cells interact with these target genes.

## 5 Conclusion

This study identified SLC27A1 and METTL17 as key genes dysregulated in CKD, with experimental validation in patient peripheral blood samples and a UUO mouse model confirming their altered expression. Proximal tubular cells and smooth muscle cells were revealed as principal sites of these genes dysregulation, and ligand–receptor interactions may mediate pathogenic cell–cell communication. Immune dysregulation involving naïve B cells, activated NK cells, and γδ T cells further contributes to disease progression. These findings provide a mechanistic framework linking metabolic, cellular, and immune alterations in CKD and suggest potential therapeutic targets.

## Data Availability

The datasets presented in this study can be found in online repositories. The names of the repository/repositories and accession number(s) can be found in the article/**Supplementary Material**.

## Supplementary material

Supplementary Figure 1 P-value distribution plot for CIBERSORT immune cell deconvolution analysis samples.

Supplementary Figure 2 Heatmaps of marker gene expression across various cell populations.

Supplementary Table 1 Marker genes for cell type annotation.

Supplementary Table 2 A total of 63 candidate genes were identified by the intersection of DEGs, MRGs, and module genes.

Supplementary Table 3 Enrichment data of 67 candidate genes in biological functions and pathways.

Supplementary Table 4 The top 15 pathways enriched by KEGG pathways.
