# Supplementary figures and images for: Integrative omics and experimental validation reveal METTL17 and SLC27A1 as biomarkers and potential therapeutic targets in chronic kidney disease
Source: Front Immunol. 2026 Feb 13;17:1724740. doi: 10.3389/fimmu.2026.1724740 (PMC12946038; doi:10.3389/fimmu.2026.1724740)

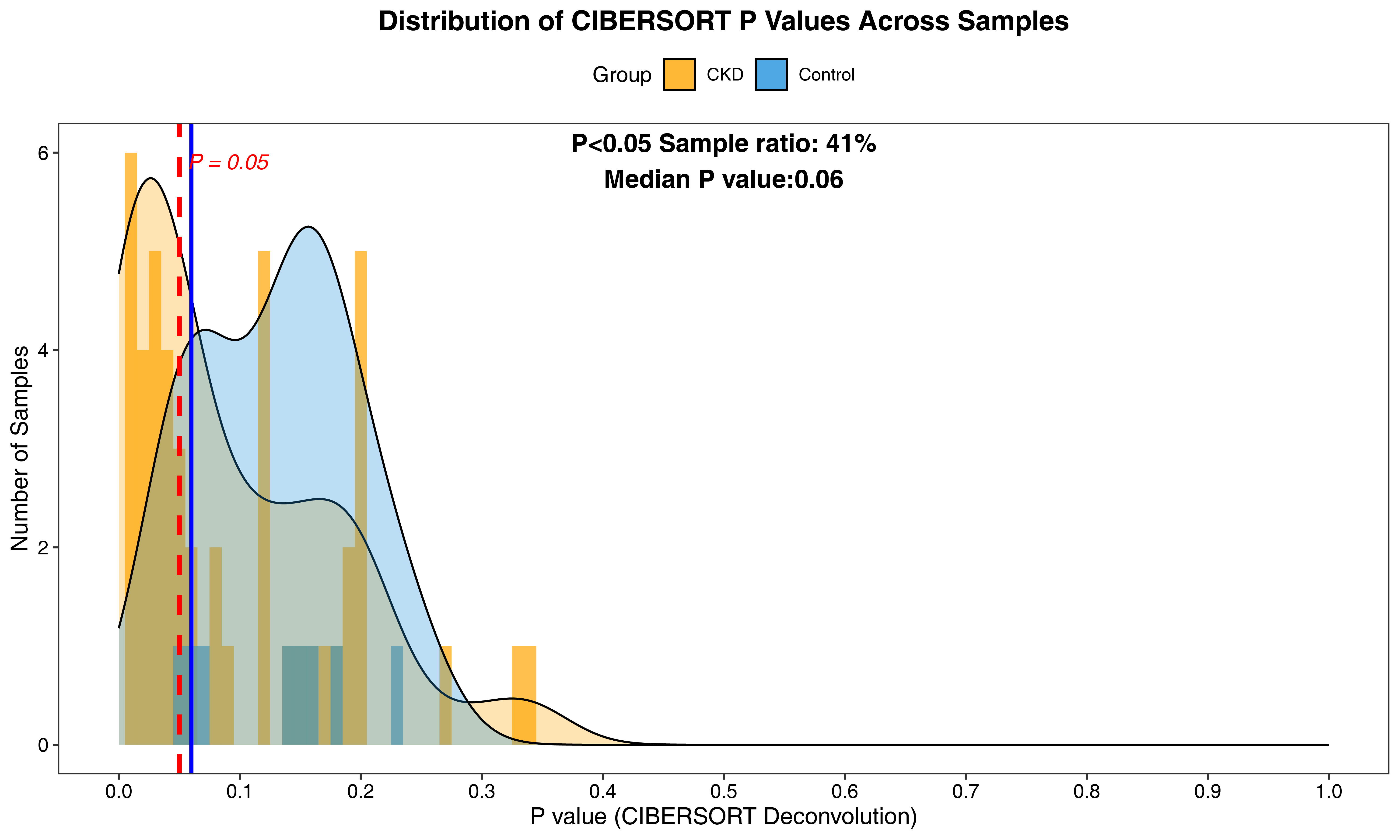

Supplement: Supplementary Figure 1 — P-value distribution plot for CIBERSORT immune cell deconvolution analysis samples. [file Image1.tif]

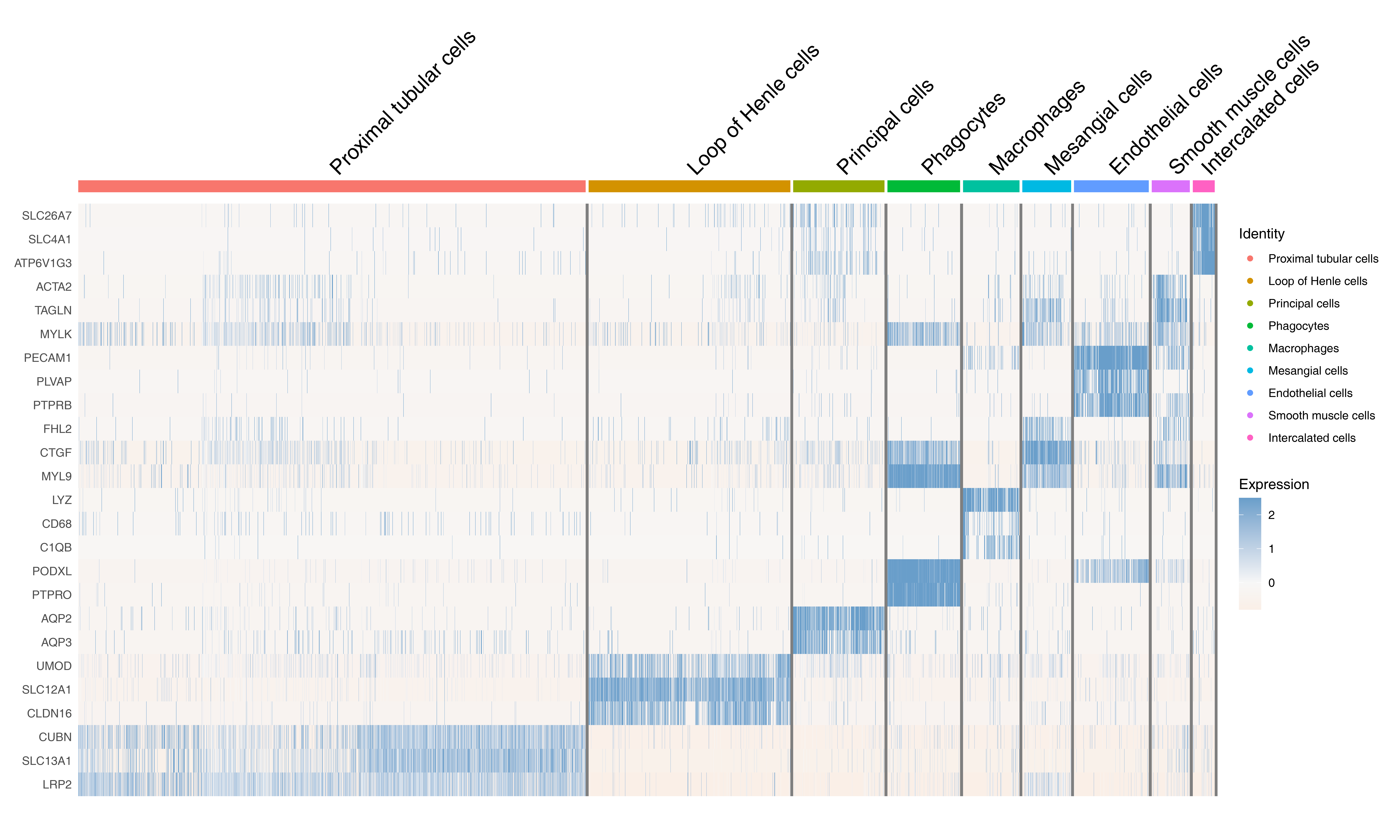

Supplement: Supplementary Figure 2 — Heatmaps of marker gene expression across various cell populations. [file Image2.tif]
