# Supplementary material for: Integrative omics and experimental validation reveal METTL17 and SLC27A1 as biomarkers and potential therapeutic targets in chronic kidney disease
Source: Front Immunol. 2026 Feb 13;17:1724740. doi: 10.3389/fimmu.2026.1724740 (PMC12946038; doi:10.3389/fimmu.2026.1724740)
Supplement: Supplementary Table 1 — Marker genes for cell type annotation. [file Table1.docx]

**Supplementary Table S1:** Marker genes for cell type annotation.

| celltype | marker |
| --- | --- |
| Intercalated cells | SLC26A7, SLC4A1, ATP6V1G3 |
| Smooth muscle cells | ACTA2, TAGLN, MYLK |
| Endothelial cells | PECAM1, PLVAP, PTPRB |
| Mesangial cells | FHL2, CTGF, MYL9 |
| Macrophages | LYZ, CD68, C1QB |
| Phagocytes | PODXL, PTPRO |
| Principal cells | AQP2, AQP3 |
| Loop of Henle cells | UMOD, SLC12A1, CLDN16 |
| Proximal tubular cells | CUBN, SLC13A1, LRP2 |
